# Pre- and post-operative voice therapy for benign vocal fold lesions: protocol for a non-randomised, multicentre feasibility trial with embedded process evaluation

**DOI:** 10.1186/s40814-024-01508-1

**Published:** 2024-05-23

**Authors:** Anna White, Paul Carding, Vicky Booth, Julian McGlashan, Jarrad Van Stan, Pip Logan, Rehab Awad

**Affiliations:** 1 Centre for Rehabilitation & Ageing Research, Academic Unit of Injury, Recovery and Inflammation Sciences, School of Medicine, University of Nottingham, Nottingham, UK; 2 Oxford Institute of Applied Health Research, Oxford, UK; 3 Nottingham University Hospitals NHS Trust, Nottingham, UK; 4 Massachusetts General Hospital, Boston, USA; 5 University Hospital Lewisham NHS Trust, London, UK; 6 Kasr Al-Aini Hospital, Cairo University, Cairo, Egypt

**Keywords:** Pre- and post-operative, Benign vocal fold lesions, Voice therapy, Phonosurgery, Feasibility, Process evaluation

## Abstract

**Background:**

Management of benign vocal fold lesions (BVFLs) is variable with individuals receiving surgery, voice therapy, or a combination of these approaches. Some evidence suggests that the best outcomes may be achieved when patients are offered pre- and post-operative voice therapy in addition to phonosurgery, but what constitutes pre- and post-operative voice therapy is poorly described. The pre- and post-operative voice therapy (PAPOV) intervention has been developed and described according to the TIDieR checklist and Rehabilitation Treatment Specification System (RTSS) for voice. The PAPOV intervention is delivered by specialist speech and language therapists trained in the intervention and comprises 7 essential and 4 additional components, delivered in voice therapy sessions with patients who are having surgery on their vocal folds for removal of BVFLs.

**Study design:**

Non-randomised, multicentre feasibility trial with embedded process evaluation.

**Method:**

Forty patients from two sites who are due to undergo phonosurgery will be recruited to receive the PAPOV intervention. Measures of feasibility, including recruitment, retention, and adherence, will be assessed. The feasibility of gathering clinical and cost effectiveness data will be measured pre-treatment, then at 3 and 6 months post-operatively. An embedded process evaluation will be undertaken to explain feasibility findings.

**Discussion:**

This study will assess the feasibility of delivering a described voice therapy intervention protocol to patients who are undergoing surgery for removal of BVFLs. Findings will be used to inform the development and implementation of a subsequent effectiveness trial, should this be feasible.

**Trial registration:**

This trial has been prospectively registered on ISRCTN (date 4th January 2023), registration number 17438192, and can be viewed here: https://www.isrctn.com/ISRCTN17438192.

## Background

Voice is a vital part of everyday human communication; however, one in three people will experience dysphonia in their lifetime [1]. The aetiology of dysphonia can be multifactorial, including muscle tension, tissue inflammation, nerve damage, or structural changes on the vocal folds [2]. Vocal fold cysts, polyps, papilloma, and Reinke’s oedema are examples of structural changes, termed ‘benign vocal fold lesions’ (BVFLs). BVFLs cause dysphonia by causing irregular vibration, preventing vocal fold closure, and increasing compensatory muscle tension [3, 4].

Voice loss because of BVFLs impacts individuals, society, and health resources. Individuals struggle to communicate on a daily basis and are unable to partake in hobbies, socialise, or fulfil caring responsibilities. Reduced activity and participation in society leads to increased rates of social isolation, anxiety, and depression [5, 6]. It is estimated that consequential time off work alone costs the UK £200 million each year [7] and direct healthcare costs in the US total £10–11 billion per year, making dysphonia comparable with COPD and diabetes [8, 9].

There are no clinical guidelines determining management for patients with BVFLs [10]. Patients may be offered surgery, pharmacological management, voice therapy, or a combination of approaches [11]. Pre-operative voice therapy can result in avoidance of surgery in up to 50% of patients with vocal fold polyps [10, 12–14]. Voice therapy may lead to lesion regression due to a reduction of phonotraumatic behaviours, improvement of throat symptoms as a result of improved voice care, or a reduction of muscle tension patterns. Emerging research suggests that voice therapy delivered by a specialist speech and language therapist (SLT) pre- and post-operatively gives greater improvement than surgery alone [15, 16]. However, it is unclear which elements of voice therapy are most effective, when they should be introduced or with which patients. In addition, treatment for individuals with BVFLs is influenced internationally by local capacity, historical practice, and surgeon preference [11, 17]. This can result in sub-optimal patient care [18], unnecessary surgery, additional hospital appointments, prolongation of symptoms [19], secondary complications from surgery [20], raised anxiety, and financial consequences [8].

Voice therapy is a complex intervention comprising multiple, interacting components [21]. To assess the effectiveness of a complex intervention, work must first be undertaken to interrogate the active ingredients in the intervention, the context in which it is delivered and the flexibility or tailoring which occurs in clinical practice. The Rehabilitation Treatment Specification System (RTSS) for voice [22] has proposed unique and specific targets and ingredients for voice therapy. The aim of this is to improve descriptions of voice therapy interventions and untangle the ‘black box’ scenario around complex interventions. Currently it can be unclear what is meant by generic treatment descriptions. The Medical Research Council’s framework for developing and evaluating complex interventions [23] advocates a structured developmental pathway by undertaking preliminary intervention development work, followed by feasibility testing. This reduces the likelihood of research waste, which they describe is an inevitable consequence of failing to devote adequate time to developing, describing, and testing a complex intervention [21, 24]. Work has now been completed to identify the potential ingredients in a best practice voice therapy intervention. This has involved the triangulation [25] of findings from a systematic review [26], expert interview study [17], national survey of current practice [27], a Delphi consensus study [28], and extensive Patient and Public Involvement and Engagement (PPIE) activities.

The outcome is a Pre- And Post-Operative Voice therapy intervention (PAPOV) which is described in detail, in accordance with the TIDieR checklist [29], and according to the Rehabilitation Treatment Specification System (RTSS) for voice [22, 30]. The PAPOV intervention has been specifically developed for adults who are undergoing phonosurgery to have BVFL(s) removed. It is hoped that patients who receive the PAPOV intervention will feel prepared for their surgery, understand how to optimise their preparation for and recovery from surgery, and develop the vocal skill necessary for long-term healthy voice management. It is now appropriate to test the feasibility of delivering this intervention to patients who are undergoing phonosurgery for removal of BVFLs.

This research will address key uncertainties in undertaking a trial of pre- and post-operative voice therapy, such as recruitment, retention, intervention fidelity, and acceptability.

## Aims and objectives

This paper describes a non-randomised multicentre feasibility trial with an embedded process evaluation. The aim of the trial is to explore feasibility elements including recruitment, retention, delivery of the voice therapy intervention, completion of clinical outcomes, and economic evaluation data. An embedded process evaluation will investigate the delivery, context, and mechanisms of impact in the PAPOV intervention, in order to explain feasibility findings.

Primary feasibility objective: The primary objective is to measure the feasibility of delivering the PAPOV intervention in a multicentre, non-randomised trial to patients undergoing phonosurgery for benign vocal fold lesions.

The feasibility trial objectives are to gather data regarding the following parameters:

1. Number of eligible patients, measured using the eligibility log and surgical lists at each site at the end of the study
2. Number of patients recruited and consented to the trial, as a proportion of those eligible, measured using the eligibility log at each site at the end of the study
3. Number of patients completing the study, as a proportion of those recruited measured using the case report form at the end of the study
4. The amount of clinical outcomes data completed, measured using the case report form at each time point (baseline, 3 and 6 months post-surgery) (%)
5. The amount of health economics data completed, measured using the case report form at each time point (baseline, 3 and 6 months post-surgery) (%)

Process evaluation objectives are:

1. Number of voice therapy sessions received by each patient measured using clinical notes at the end of the study
2. Level of adherence to the PAPOV intervention within voice therapy sessions measured as the number of essential components documented in clinical notes for each session
3. Description of any adaptations or alterations made to the PAPOV intervention measured using analysis of clinical notes for each session and analysis of clinician interviews
4. The amount and completeness of home practice measured using adherence questionnaire data completed by participants at the end of their voice therapy sessions
5. Description of participating sites and participants, measured using the case report form at the end of the study
6. Understand clinicians’ experiences of being trained to deliver the intervention and trial processes, measured using an analysis of clinician mentoring records and interview data with clinicians at the end of the study
7. Understand clinicians’ experiences of delivering the intervention including acceptability, barriers, and facilitators measured using analysis of clinician mentoring records and interview data with clinicians at the end of the study
8. Understand participants’ experiences of trial processes measured using analysis of interview data at 6 months post-surgery
9. Understand participants’ experiences of receiving the PAPOV intervention including acceptability, barriers, and facilitators measured using analysis of interview data at 6 months

## Methods

### Study design

This is a multicentre, non-randomised, feasibility trial with an embedded process evaluation.

Problems associated with acceptability, compliance, delivery of the intervention, recruitment, retention, and effect sizes can occur if preparatory work is insufficient when evaluating the effectiveness in a definitive RCT [24]. The mixed methods design permits the depth of data collection and analysis required to inform changes in a definitive trial. The multicentre design will permit additional feasibility parameters to be gathered. This protocol follows guidance in the ‘CONSORT 2010 statement: Extension to randomised pilot and feasibility trials’ [31]. Process evaluation is an essential part of designing and testing complex interventions. Investigating the implementation of the intervention, including the fidelity, dose, adaptations, and reach, along with the context and mechanisms of impact informs interpretations of the outcomes of the trial. The Medical Research Council recommends including process evaluation at the feasibility stage, to understand and interpret feasibility findings and inform further development and evaluation stages [23, 32].

### Participants

**Fig. 1:**
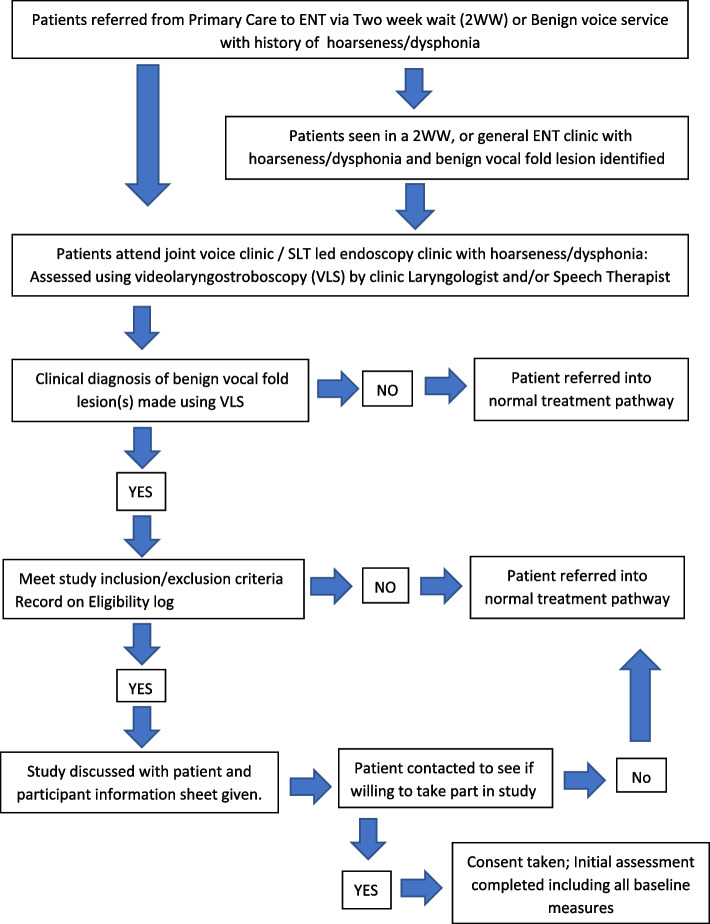
Eligibility and screening flow diagram

Patients with BVFLs who have been consented for phonosurgery by an Ear Nose and Throat (ENT) surgeon as part of their management will be eligible for enrolment. Table 1 lists the inclusion and exclusion criteria. Fig. 1 shows a flow diagram detailing eligibility and screening procedures.

**Table 1:** Inclusion and exclusion criteria

Inclusion criteria
• ≥ 18 years of age
• Willing and able to offer informed consent to the study
• Presence of unilateral or bilateral benign vocal fold lesion on the vibrating portion of the vocal fold (including one or a combination of these diagnostic categories: fibrotic vocal fold nodules, polyp, cyst, pseudocyst, polypoid fringe, sulcus, mucosal bridge, papilloma)
• Clinical diagnosis confirmed using video laryngostroboscopy
• ± presence of additional muscle tension dysphonia/inflammation
Exclusion criteria
• Diagnosis of soft vocal fold nodules; these patients receive a different pathway of care and surgery would not be offered as first line treatment
• Diagnosis of arytenoid granuloma; this does not affect the vibratory portion of the vocal fold
• Previous phonosurgery due to the potential for scarring
• Suspicion of malignancy requiring urgent microlaryngoscopy and biopsy; principles of phonosurgery for malignancy are different from benign lesions

### Sampling

We aim to recruit 40 participants from 2 sites, aiming for between 15 and 25 per site. Additionally, clinicians delivering the intervention will be invited to take part in interviews (*n* = 4–6).

Sample sizes of between 24 and 50 participants are recommended to give sufficient information to estimate key parameters in feasibility trials [33, 34]. This trial aims to recruit 40 participants over 2 sites. Audit data from Nottingham confirms surgical data of over 70 eligible patients per annum with other centres reporting comparable figures, meaning it will take an estimated 7 months to recruit 20 patients if a conservative recruitment rate of 50% is achieved [35]. Regardless of the number of enrolled participants, recruitment will stop after 10 months on each site, owing to the timescales for the project. Data from a systematic review of functional voice therapy reported attrition rates of 0–39% meaning that as a minimum, > 24 participants are expected to complete the intervention.

### Sampling technique

Participants will be identified from ENT and SLT clinics in two NHS trusts. A third NHS trust will be registered and opened in the event of poor recruitment or difficulties with capacity of clinicians or surgical waiting lists. All trusts have confirmed their desire to participate, have research active clinicians improving their capability to participate in a feasibility trial, and have well-established voice services with sufficient patient numbers to fulfil recruitment target.

### Recruitment

The study will be publicised to all ENT and SLT clinicians within the participating departments. All patients presenting to the Joint Voice Clinic in the ENT department at Queens Medical Centre campus, Nottingham University Hospitals Trust, and Lewisham and Greenwich NHS Trust with a diagnosis of a benign vocal fold lesion, and who have been consented for surgery will be considered. Patients presenting to other types of ENT clinics at these sites (General ENT clinic, 2WW clinic) will also be considered on the proviso that they meet the full inclusion criteria. In addition, patients meeting the inclusion criteria with a diagnosis of a BVFL who are on the waiting list for phonosurgery will be invited to take part in the study. Any individual who presents with a BVFL will be screened for eligibility and documented in a screening log. The number of those eligible as a proportion of those screened will be reported alongside reasons for screening failures in order to understand the presenting populations more fully.

### Sample identification

Information about the trial will be on display in the relevant clinical areas, in the form of a patient poster and clinician poster. In most instances, the initial approach will be from a member of the patient’s usual care team, face-to-face during a standard clinic appointment. Clinicians will give eligible participants who express an interest, an information sheet and consent form.

In some instances (for example if a patient is on the waiting list prior to the opening of the study, or if the patient is noted to meet the eligibility criteria after their appointment), the treating clinician will send eligible participants a letter informing them about the study. This letter explains that they may be eligible for a research project and will include a participant information sheet. The letter will invite the patient to make contact and will state that the treating clinician will give them a courtesy call 1–2 weeks later to see if the patient would like further information.

In both instances, the clinician will seek the patient’s permission for the research team to contact the patient by phone. The research team will contact the participant by phone and if willing, set up a research clinic appointment at least 24 h after the participant information sheet (PIS) has been given, to take consent and gather baseline data.

If needed, the usual hospital interpreter and translator services will be available to assist with discussion of the trial, the PISs, and consent forms. However, the consent forms and information sheets will not be available printed in other languages.

### Ethics and consent

This study has received a favourable ethical opinion by the Health Research Authority (HRA) and Health and Care Research Wales (HCRW) and West London and GTAC Research Ethics Committee (Reference no 22/LO/0859).

Participants who have been identified by their clinical teams as potentially eligible and have given permission will be contacted by the research team. Those who are willing to take part will be invited to a virtual research appointment using an NHS-approved telehealth platform. Participants will provide informed consent at this appointment.

If a participant is unable to access the virtual research appointment, they will be invited to a face-to-face appointment at their treating hospital where they will have the opportunity to discuss the trial and give informed consented face-to-face.

Informed consent will be collected from each participant before they undergo any interventions related to the study. A participant’s involvement in the study will discontinue at their request. Participants will not be prevented from taking part in the study if they are receiving additional therapeutic intervention, e.g. physiotherapy, and they wish to take part in this trial. However, participation in concomitant behavioural interventions will be noted at recruitment, to monitor the potential level of burden on the patient.

As part of the process evaluation, all clinicians who have delivered the PAPOV intervention will be invited to take part in an interview to talk about the intervention and their experiences of being involved in a trial. Clinicians will be given an information sheet and consent form, and it will be explained to them that their involvement in the interviews is optional. Consent will be taken at the start of the interview, or prior to the interview, depending on the wishes of the clinician.

### Intervention

**Fig. 2:**
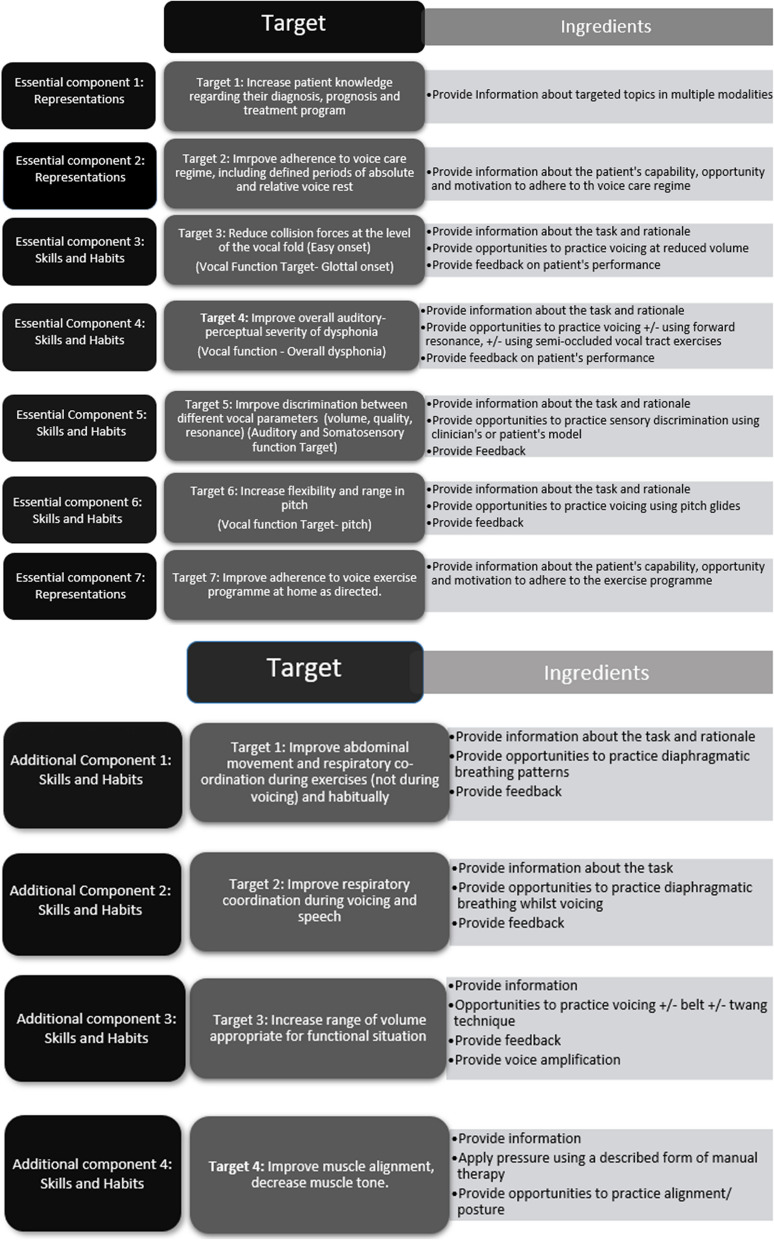
Essential and additional components of the PAPOV intervention showing targets and ingredients

The pre- and post-operative voice therapy intervention (PAPOV) will be delivered by specialist voice therapists, trained in the intervention at the participant’s treating hospital. PAPOV comprises 7 essential and 4 optional treatment components and includes a balance of information, advice, education, and practical vocal exercises (see Fig. 2). Voice therapy sessions will either be done face to face at the hospital or via a hospital-approved video link. Participants will have a minimum of two sessions of voice therapy before their operation. In these pre-operative voice therapy appointments, participants will be given information and advice about their voice, diagnosis, and surgery. They will develop voice-related goals and will be taught voice exercises to help produce their voice in a healthy way.

The participant will come into the hospital for surgery to remove the lesion from their vocal fold. The timing of this will depend on waiting lists for surgery at the treating hospital. All details about the surgery and the participants’ time in hospital will be given to them by their treating ENT team. Each participant will receive a follow-up appointment with their ENT doctor as per routine care to assess voice recovery, check healing of the vocal folds, and discuss histology results and the outcome of the surgery. This will be done 6–8 weeks after the date of surgery.

**Fig. 3:**
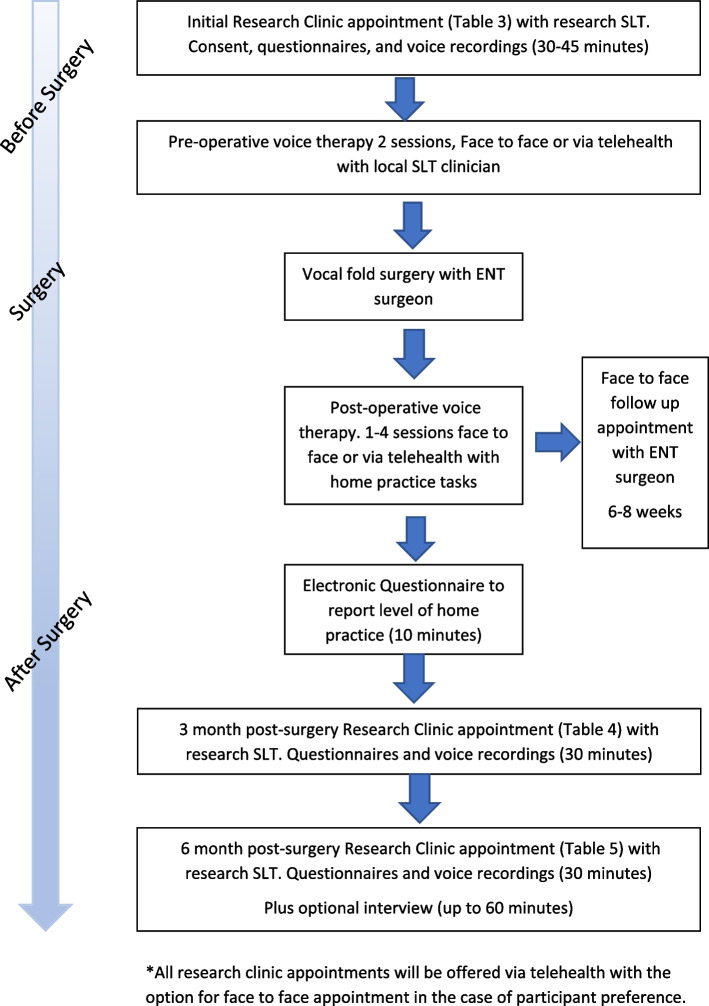
Participant flow chart

Post-operative voice therapy sessions will recommence approximately 10–14 days after phonosurgery. The number of sessions will vary depending on how the participant is recovering, but each participant is likely to be offered between 1 and 4 post-operative sessions. These sessions will be done either face-to-face or via video link. The aim is to support the participant in their recovery after their operation and to help improve the sound, strength, and stamina of their voice, together with return of functional voice activities. Participant activities are summarised in Fig. 3.

## Data collection

Consideration has been given to ensure that data collected address each of the study objectives and relate to both feasibility and process evaluation considerations. Data collection comprises primary feasibility outcomes, clinical outcome measures, economic evaluation, and intervention fidelity and acceptability. All measures have been reviewed, discussed, agreed, and piloted by the patient and public involvement (PPI) group and are discussed in the section below.

### Primary feasibility outcomes

Recruitment: The number of patients recruited to the trial as a proportion of those eligible will be measured at each site. Audit data from site one confirmed surgical data of over 70 eligible patients per annum, meaning it will take 7 months to recruit 20 participants per site, if a conservative recruitment rate of 50% is achieved. Recruitment figures will be monitored on a monthly basis at the trial management group and a decision to open a third site will be triggered if recruitment falls below 50% of the target after 4 months.

Retention: The number of patients completing the study as a proportion of those recruited will be measured. Data from a systematic review of functional voice therapy reported attrition rates of 0–39%. Attrition rates will therefore be considered acceptable if they remain below 39%. Reasons for attrition will be considered. These will specifically be explored from both the clinician perspective and the participant perspective using semi-structured interviews.

Data completion: The amount (%) of clinical and health economics data completed will be measured at each timepoint (Baseline, 3 and 6 months post-surgery). Data checks will be undertaken throughout to identify if there is missing data and the reasons for this. Steps to address this will be implemented as required during the trial, such as the provision of additional technological support, participant reminders, offering face to face appointments for data completion.

### Secondary patient-centred outcomes

#### Schedule of data collection

Participants will attend 3 virtual or face to face research clinics. These will be scheduled at enrolment (pre-operatively), then at 3 and 6 months post-operatively to gather outcomes data. Participants will also be asked to complete a short adherence questionnaire online, or on paper according to patient preference. This will be sent to the participant when their therapist has confirmed completion of the PAPOV intervention. Participants who gave additional consent to be interviewed will be contacted 6 months post-surgery to talk about their experiences of the trial process and intervention acceptability. All clinicians who have been involved in delivering the intervention and who have given informed consent will also be contacted and interviewed (see Fig. 2).

#### Clinical outcome measures

These outcome measures have been carefully selected to provide a multidimensional assessment of the voice, following a review of the current voice disorders literature, and discussion with patients and clinicians.

Patient-reported outcome measures.

1. Voice Handicap Index-10 [36]: The VHI-10 is a validated patient-reported outcome measure covering physical, functional, and emotional aspects of voice. This is likely to be the primary outcome in a full trial. The VHI-10 is a shortened version of the VHI-30 [37], which makes it a quick and easy self-administered tool with no loss of utility of validity compared to the VHI. Scores can range from 0 (no handicap) to 40 (maximal handicap). A score greater than 11 is considered abnormal [38] and a change of 6 points over time represents a minimally important difference [39].
2. Reflux Symptom Index (RSI) [40]: The RSI is a validated 9-point patient-completed questionnaire targeting symptoms associated with laryngopharyngeal reflux (LPR). The association between RSI and VHI improvement indicates good construct validity [40]. Basic science studies suggest that laryngopharyngeal reflux creates an environment that may predispose individuals to BVFLs because of changes in defence mechanisms of the vocal folds, cell to cell dehiscence, inflammatory reaction of the vocal folds, and reaction to phonotrauma [41] and is therefore of particular relevance to this patient group. Patients are asked to rate the severity with which they experience different symptoms on a scale of 0 (no problem) to 5 (severe problem). Scores can range from 0 to 45, with a score over 13 considered to be abnormal [42]. Information about pharmacological treatment for laryngopharyngeal reflux, including any changes in medication, will be gathered at research clinic to provide additional context for RSI scores.
3. Vocal Tract Discomfort Scale (VTDS): The VTDS is a self-administrated questionnaire designed to measure the subjective perception of sensory discomfort in the throat (vocal tract) [43]. Although patients may report a change in voice quality, sensory symptoms are also frequently present [44]. There is a difference between the reported levels of discomfort depending on the cause of the voice problem. Patients with BVFLs were found to have higher scores on the VTDS than other types of voice disorders [44]. Therefore, it is of particular importance that these symptoms are monitored. The VTDS asks the patient to rate the frequency of occurrence and severity of eight subjectively different sensations: burning, tightness, dryness, aching, tickling, soreness, irritability, and lump in the throat. The frequency and severity are rated separately on a 7-point Likert scale ranging from 0 to 6 for frequency (0 = never, 2 = sometimes, 4 = often, 6 = always) and for severity (0 = none, 2 = mild, 4 = moderate, 6 = extreme). VTDS scores have been shown to correlate with the total scores of the VHI and decrease after voice training and vocal hygiene education in teachers [45]. A change in the Persian version of the VTDS of 6.0 points for each subscale following a therapeutic intervention has been interpreted as a real change with a 95% confidence level [46]; however, this is in patients with muscle tension dysphonia. Pre- and post-operative changes have not been studied to our knowledge.

Clinician-rated outcome measures.

1. Laryngostroboscopic video examination: All patients will have a laryngostroboscopic (flexible nasoendoscopy or rigid transoral) examination prior to recruitment to confirm the presence of benign vocal fold lesion(s) and diagnose the lesion type. This will be repeated at a 6–8-week post-operative ENT review. Stroboscopy data is gathered routinely post-operatively as part of usual care. This will inform the timing of this outcome in a definitive trial. Video laryngostroboscopic images will be used for blinded analysis by laryngologists and SLTs to rate a series of parameters including vocal fold appearance, closure pattern, glottal symmetry, vocal fold movement, and mucosal wave. Two rating scales have been modified from published scales [47, 48] and replicate the scales used in a recently published paper [49].
2. High-quality voice recording: The participant will be given instructions and helped to download the freely available app ‘Voice Record Pro’ (Developer Dayana Networks Ltd) on to a smartphone or tablet. Participants will be supported follow a written voice tasks protocol, holding their phone at a distance of 10 cm from their mouth, and using standardised recording settings. A recording will then be made of the participant’s voice whilst (a) sustaining a vowel sound /a/, (b) reading a standardised passage of text aloud (The north wind and the sun), (c) performing three repetitions of a pitch glide from lowest to highest note, and (d) performing three repetitions of maximum phonation time (MPT) on an /a/ sound. The file will be saved on the participants’ phone/tablet and immediately emailed to the research team. The file will be saved to a secure drive and the email deleted. This recording will be used to undertake perceptual (CAPE-V), aerodynamic, and acoustic analysis described below.
3. CAPE-V: The Consensus Perceptual Auditory Evaluation of Voice (CAPE-V) is a validated auditory-perceptual measurement of voice, rated by a clinician [50, 51]. It provides an overall rating of severity as well as discreet ratings of specific vocal parameters including roughness, breathiness, strain, pitch, and loudness. In this study, pitch and loudness will be omitted. These parameters can be assessed more reliably with acoustic measures and the omission will reduce rater fatigue.

Three speech and language therapists experienced in perceptual analysis will assess a sustained vowel and the reading passage ‘The north wind and the sun’. Raters will be blinded to the stage of treatment and will not have treated the participant. Raters will receive a brief refresher training programme in the use of CAPE-V to improve inter-rater reliability and will have external anchor voices provided to overcome the reduced intra-rater and inter-rater agreement associated with the increased freedom of judgement [52]. The mean rating of the raters for each recording will be used as the data point for individual patients.

b) Aerodynamic assessment of voice: Maximum phonation time (MPT) is a simple aerodynamic voice parameter for measuring glottal competence. The patient is asked to take a deep breath and sustain a steady ‘ah’ vowel sound as in ‘far’ or ‘car’ for as long as possible. MPT is measured in seconds. MPT has potential value with this population due to the frequent impact of the vocal fold lesion impacting on closure of the vocal folds. The increased air escape often results in a reduced MPT. Therefore, it is hypothesised that MPT will increase post-operatively.
c) Acoustic analysis data: Acoustic measures of voice provide and objective assessment of vocal function. Participants will record their voice using the Voice Record Pro app, save this as an uncompressed.wav file, and email it to the research team for analysis, using the software VOXPlot ©lingphon/Jorg Mayer. VOXPlot is an open source, freely available tool for the analysis of 18 acoustic parameters slope (dB), tilt (dB), HF noise (dB), HNR-D (dB), H1H2 (dB), CPPS (dB), jitter local (%), jitter ppq5 (%), shimmer (%), shimmer (dB), HNR (dB), PSD (ms), voice breaks, GNE, pitch mean (Hz), pitch min (Hz), pitch max (Hz), pitch sd (Hz), pitch range (st), and two multidimensional indices (AVQI and ABI). Analysis results are presented as a voice profile which contains examination data, numerical analysis results of the vowel sample (incl. norm values), a narrowband spectrogram of the vowel sample, and a norm-value circle that highlights deviations of the vowel sample in 12 acoustic dimensions using traffic light system (norm range: green/deviation: red) [53]. Comparison of pre- and post-operative voice recordings will be undertaken. It is hypothesised that an improvement in acoustic properties of the voice will be seen at the primary end point (6 months post-operatively).

### Economic evaluation measures

Early involvement of economic evaluation is recommended by the Medical Research Council [23] and considered an essential component of feasibility testing by some [54]. One objective of feasibility studies is to define and refine methods for data collection [54]. This includes health economics data. Economic evaluation is necessary if implementation into standard care is anticipated. Although some attempts have been made to consider costs associated with dysphonia [8, 9], there is a lack of health economics data within the voice disorders literature, and certainly within the literature on management of BVFLs.

1. Voice-Related Quality of Life [55]. The VR-QOL is a patient-reported, disease specific questionnaire for voice disorders. The 10-item questionnaire has been shown to be valid, reliable, and responsive to change in patients with dysphonia of varying aetiologies. Patients are asked to rate on a scale from 1 (none, not a problem) to 5 (problem is as ‘bad as it can be’) 10 statements about their voice over the previous 2 weeks. The inclusion of a disease specific quality of life measure to inform future measures of quality-adjusted life years (QALYs) is recommended [54].
2. EQ-5D-5L [56]: The EQ-5D is a family of instruments to describe and value health. Patients are asked to rate five dimensions of health: mobility, self-care, usual activities, pain/discomfort, and anxiety/depression. The EQ-5D-5L was developed to improve sensitivity and to standardise language across the dimensions and gives patients five levels for each dimension rather than the previous three [56]. Evidence obtained from generic measures can be used to compare both the impact of health problems and the benefits offered by treatments across different patient populations and disease areas. Evaluation of the EQ-ED-5L can allow for a calculation of QALYs. Differences in scores can be used to show differences that may emerge in a future randomised controlled trial (RCT) [54]. It is unclear whether the EQ-5D-5L will be sensitive enough to show change in this population but as this has not been explored, it is important to understand whether this has potential value in a future trial.
3. Voice costs questionnaire (VCC): The voice costs questionnaire is an unvalidated measure, developed by the authors in conjunction with patients with lived experience of voice disorders to capture the costs of living with a voice disorder and the associated treatment received. It has four sections: (1) impact on work, (2) hospital appointments, (3) medicines, and (4) other associated costs.

### Intervention fidelity and acceptability

1. Participant compliance: The number of sessions of voice therapy offered and accepted will be individualised according to participant need. Therefore, there will be no required minimum number of voice therapy sessions attended in order to meet compliance. However, the number of pre- and post-operative sessions delivered will be documented. Understanding the number and intensity of sessions received is an important process evaluation consideration [32] and will provide valuable information regarding individual tailoring of the PAPOV intervention.
2. Participant adherence to the PAPOV intervention: Participants will complete an online adherence questionnaire as one treatment fidelity measure [57]. Understanding the level of patient adherence to the intervention both within and between sessions is fundamental to interpreting clinical outcomes data. Trialling and testing measures for evaluating adherence at a feasibility stage will inform measures to be used in future research. To overcome the limitations of individual measures, multiple measures of engagement and fidelity are recommended [58–60]. Therefore, a battery of adherence measures for the PAPOV trial have been agreed with PPI discussion.
3. Exercise Adherence Rating Scale (Beinart et al., 2016); EARS enables the measurement of adherence to prescribed home exercise using a patient-completed 6-item measure. Participants use a 5-point Likert scale to rate the extent to which they agree or disagree with a given statement, e.g. ‘I do my exercises as often as recommended’. Participants who receive the PAPOV intervention will be asked to practise exercises between sessions, and transference of exercises from the clinic to functional situations is thought to have a significant impact on voice outcomes. Although EARS has been validated on patients undertaking physiotherapy rather than voice therapy, no validated measures exist in the English language for home exercise adherence to voice therapy exercises. The measure has been reviewed by both patients and clinicians for relevance, acceptability, and ease of completion and therefore will be included.
4. Pre- and post-operative voice therapy (PAPOV) adherence questionnaire. PAPOV is a novel intervention with multiple elements. This questionnaire has been developed in accordance with guidance for developing quality fidelity and engagement measures for complex health interventions [61]. Adherence is known to be influenced by a number of factors including self-efficacy and a patient’s understanding of task rationale [62]. Participants are asked four questions regarding elements of the intervention: (1) Was X part of your treatment? (2) How relevant or important did you feel this was as part of your treatment? (3) To what extent did you follow/complete X? (4) How easy was it to stick to the advice/exercise?
5. Voice Therapy Self-Efficacy Scale (VTSES) (Van Leer 2021) Part 2 of a self-efficacy measure developed by Van Leer and Conner (2015) will be used to consider generalisation of the skills developed in therapy to intentional implementation in daily communication.
6. Clinician adherence to the intervention

The content of the intervention is outlined in Fig. 2 and described fully in the PAPOV clinician manual. All clinicians delivering the intervention will be trained in its content and delivery. There is the expectation that individual tailoring will be required according to patient need. A proportion of clinician notes will be reviewed (including at least one session from all participants and examples of all treating clinicians) to analyse whether voice therapy sessions cover the core components of the intervention. Ten percent of sessions will be videoed for analysis of intervention content to allow for cross comparison with clinicians’ notes. Analysis of notes will be undertaken throughout the trial period to enable steps to be put in place to improve intervention fidelity if required. Steps would include increasing the level of mentoring to clinicians and increasing video recording of sessions to determine whether reporting of intervention delivery in the clinical notes reflects clinician practice in sessions.

**Fig. 4:**
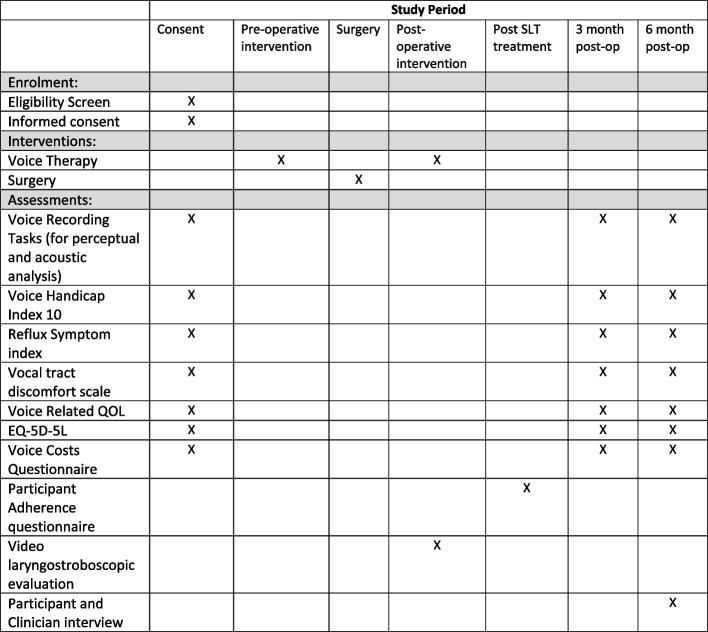
Schedule of enrolment, interventions, and assessments. *Video laryngostroboscopy will also be carried out prior to consent to ensure that the participant is eligible for the study

Fig. 4 shows a summary of the schedule of enrolment, interventions, and assessments.

## Monitoring

The chief investigator (VB) has overall responsibility for the study and shall oversee all study management. A trial management group (AW, VB, PC, PL) will meet monthly. AW will co-ordinate ethical and governance processes and undertake all aspects of study design, conduct and write up with support from the trial management group. AW will also co-ordinate clinicians in feasibility trial sites and PPI members, managing relationships and meetings with local, national, and international collaborators.

A Trial Steering Committee, comprising the chief investigator (VB), lead researcher (AW), a PPI member (DB), a laryngologist (JM), and an independent, external researcher with experience in feasibility trials (BS), will meet every 6 months during the trial to ensure that the trial is being conducted properly. A patient and public involvement group with four core members and additional contributors has contributed to all aspect of trial design, materials, and choice of outcome measures. Ongoing PPI involvement throughout the trial, including participation in the TSC and analysis of interview data, in addition to creation of dissemination materials is planned.

### Protocol compliance

Protocol deviations, non-compliances, or breaches are departures from the approved protocol. The number of sessions of voice therapy offered and accepted will be individualised according to participant need. Therefore, there will be no required minimum number of voice therapy sessions attended overall in order to meet compliance. Participants will complete an online adherence questionnaire to assess their level of compliance with the home programme advised and to give information about treatment fidelity. Clinician compliance will be monitored by analysis of written clinical notes and video footage as outlined above.

Accidental protocol deviations can happen at any time. Any deviation must be adequately documented on the relevant forms and reported to the chief investigator and sponsor immediately. Deviations from the protocol which are found to frequently recur will require immediate action and could potentially be classified as a serious breach.

### Amendments

It is the sponsor’s responsibility to decide whether an amendment is substantial or non-substantial for the purposes of submission to the Research Ethics Committee (REC). If the sponsor wishes to make a substantial amendment, the sponsor must submit a valid amendment tool to the REC for consideration. Site research and development (R&D) departments will also need to be provided with information on the amendment in order to assess their continued capacity and capability. The Health Research Authority and site R&D departments will also be notified of non-substantial amendments with participating sites assessing whether the amendment affects the continued capacity for that site.

Any amendments (substantial or non-substantial) considered necessary by the study team will only be made after discussion with the R&D department of the sponsor. Approved amendments will be reflected in an update in the protocol version.

### Assessment of risk

The level of risk to participants in this trial is deemed low. Participants will be taking part in a therapy intervention based around indirect (education and advice) and direct (vocal exercises) therapy. Participants are given instructions to monitor and throat sensations and will be seen at regular intervals to discuss any symptoms. Some discomfort in the throat is anticipated post-operatively as patients will have surgery to the voice box. The surgical procedure does not form part of the study intervention.

Specific examples of symptoms which are expected to fluctuate during the course of the study include:

- Increased level of dysphonia (more hoarse, breathy, croaky) following surgery
- Increased sensory symptoms following surgery such as soreness and aching in the throat/neck

Participants are asked about their symptoms at each appointment. These are recorded and reviewed by the study team.

All adverse events or reactions will be reported to the chief investigator who will assess and document the severity of the event and follow written procedures, including escalating to the sponsor or discussion at trial steering committees, as appropriate.

Participants are also encouraged to report any concerns regarding the trial to the chief investigator. Contact details are on the PIS.

### Data management

All investigators and study site staff will comply with the requirements of the General Data Protection Regulation 2018 and Data Protection Act 2018 with regard to the collection, storage, processing, and disclosure of personal information and will uphold the Regulation’s/Act’s core principles. All information about participants will be handled in confidence and in accordance with Nottingham University Hospital’s Research Code of Conduct and Research Ethics and Caldicott principles. Case report forms (CRFs) will only collect the minimum required information for the purposes of the trial. CRFs will be held securely, in a locked room, or locked cupboard or cabinet. Access to the information will be limited to the trial staff and investigators and relevant regulatory authorities. Computer held data including the trial database will be held securely and password protected on the Nottingham University Hospitals NHS Trust network.

All participants will be given a study number on recruitment which will be used for all data analysis. An approved transcription service will transcribe interviews with participants and clinicians. All transcriptions will be pseudonymised for any names or places. Participants will provide optional consent to be contacted with a summary of the results after termination of the study. Specific pseudonymised data will be transferred to the University of Nottingham for analysis under the conditions of a Data Transfer Agreement.

Study specific data will be stored for a period of 5 years after the study has ended. Clinical assessment and therapeutic details and outcome data will be stored in accordance with the local NHS Trust’s policy.

### Criteria for terminating trial

The sponsor and funder reserve the right to temporarily suspend or discontinue this study at any time for failure to meet expected recruitment goals, for safety or any other administrative reason. The sponsor and funder shall take advice from the trial management group and trial steering group as appropriate in making this decision. Should the study be terminated, the research data will not be destroyed.

## Analysis plan

Analysis of quantitative data will be undertaken by the CDRF (AW) with support from the supervisory team (VB, PC, PL) and University of Nottingham medical statisticians if required.

Ongoing analysis throughout the trial will be undertaken to act as an early warning system to identify early problems with the primary feasibility outcomes including recruitment, retention, or data collection which may be able to be addressed and managed. Within trial monitoring, during monthly Trial Committee meetings and six monthly Trial Steering Committee meetings will consider whether attrition may be due to administrative processes which could be improved during the trial. We shall follow approved process for dealing with non-substantial and substantial amendments in this study. Any amendments (substantial or non-substantial) considered necessary by the study team will only be made after discussion with the Trial Steering Committee and Research and Innovation department of the sponsor at NUH. Approved amendments will be reflected in an update in the protocol version.

On study completion, descriptive statistics will be used to report the proportion of eligible patients recruited, the proportion of patients who complete the study, and the proportion of patients completing the intervention per protocol. Clinical outcomes, measured as degree of change between baseline and primary endpoint (6 months), will inform sample size calculations for a future definitive trial and consider whether the outcomes chosen are likely to show both statistically and clinically significant differences. The amount of missing data at each time point will be recorded to assess the feasibility of using each measure in a future effectiveness trial. SPSS [63] will be used to manage quantitative data and perform statistical analyses.

Qualitative analysis will be undertaken by the CDRF with support from the supervisory team. Analysis of interview data will be conducted using the framework technique, a type of thematic analysis, building on the work of Miles and Huberman [64]. The framework will be developed based on the COM-B model of behaviour change, considering barriers and facilitators to the intervention and research processes [65]. An appropriately trained PPI member will undertake secondary analysis of a proportion of the participant interview transcripts. Nvivo software will be used to facilitate stages of the data management and analysis process.

### Trial progression

The trial will be deemed successful based on the following:

- Recruitment: Recruitment of > 50% of eligible participants into the trial
- Retention: Participant attrition below 39%, based on the number of participants completing the trial as a proportion of those consented

Modifications should be made to a future trial design if feasibility outcomes fall below the above criteria. Analysis of intervention fidelity data, participant and clinician interviews will inform the need for any modifications to the intervention in a subsequent trial.

## Dissemination

The dissemination strategy and proposed outputs aim to reach patients, public, clinicians, managers, and commissioners, provide training opportunities, and enhance international collaborations for future research. This includes:

- A video explaining research background and findings; produced for members of the public and health professionals and shared using social media and voice support charities.
- A publication in peer-reviewed journals to ensure clinicians worldwide can interpret and apply the findings.
- Presentation of findings at national and international clinical conferences.
- Discussion of clinical impact at multidisciplinary forums.
- A poster summarising findings, developed with PPI members, will be displayed in waiting areas and circulated to ENT departments nationally and internationally.
- A reusable learning tool, featuring PPI members, will be produced, to prepare patients for surgery (shared with ENT departments and on social media).

## Discussion

This study will assess the feasibility of delivering a described voice therapy intervention protocol to patients who are undergoing surgery for removal of benign vocal fold lesions (BVFLs). Voice therapy effectiveness studies often suffer from a lack of explicit description of the intervention delivered, making it difficult to identify what components of the therapy may have contributed to the results reported. Considerable intervention development work has been undertaken, involving a wide range of stakeholders to ensure that this intervention is fully described. The intervention will be delivered by multiple therapists across two sites strengthening the research design and generalisability of findings. All people enrolled in this study will receive training in the PAPOV intervention. A limitation of this non-randomised approach is that the randomisation method for application in any subsequent RCT cannot be tested.

Feasibility will consider both trial processes and adherence to the intervention. The embedded process evaluation will enable deeper exploration of the factors contributing to the results obtained. Findings will inform the development and implementation of a subsequent effectiveness trial, should this be feasible.

## Data Availability

The final anonymised trial dataset will be available to other researchers on request from the corresponding author.
