# Correction: Pre‑ and post‑operative voice therapy for benign vocal fold lesions: protocol for a non‑randomised, multicentre feasibility trial with embedded process evaluation

**DOI:** 10.1186/s40814-024-01572-7

**Published:** 2024-11-19

**Authors:** Anna White, Paul Carding, Vicky Booth, Julian McGlashan, Jarrad Van Stan, Pip Logan, Rehab Awad

**Affiliations:** 1 Centre for Rehabilitation & Ageing Research, Academic Unit of Injury, Recovery and Inflammation Sciences, School of Medicine, University of Nottingham, Nottingham, UK; 2 Oxford Institute of Applied Health Research, Oxford, UK; 3 Nottingham University Hospitals NHS Trust, Nottingham, UK; 4 Massachusetts General Hospital, Boston, USA; 5 University Hospital Lewisham NHS Trust, London, UK; 6 Kasr Al-Aini Hospital, Cairo University, Cairo, Egypt

**Correction** **:** **Pilot Feasibility Stud 10, 84 (2024)**

**https://doi.org/10.1186/s40814-024–01508-1**

Following publication of the original article [1], the authors would like to add an additional statement to the Acknowledgement section. The additional statement have been highlighted in **bold typeface**.

**Acknowledgements**

We thank all the patients in the patient and public involvement group for their invaluable contributions and insights: Dan, Caroline, Farzana, Deb, and Helen. **Dr Van Stan's work on this article was supported by the National Institute on Deafness and Other Communication Disorders Research Grant R01 DC020247 (principal investigator: Jarrad Van Stan). The article’s contents are solely the responsibility of the authors and do not necessarily represent the official views of the National Institutes of Health.**

The original article [1] has been updated.
